# Adult *Camk2a* gene reinstatement restores the learning and plasticity deficits of *Camk2a* knockout mice

**DOI:** 10.1016/j.isci.2022.105303

**Published:** 2022-10-08

**Authors:** Pomme M.F. Rigter, Ilse Wallaard, Mehrnoush Aghadavoud Jolfaei, Jenina Kingma, Laura Post, Minetta Elgersma, Ype Elgersma, Geeske M. van Woerden

**Affiliations:** 1 Department of Clinical Genetics, Erasmus Medical Center, 3015GD Rotterdam, the Netherlands; 2 ENCORE Expertise Centre for Neurodevelopmental Disorders, Erasmus Medical Center, 3015GD Rotterdam, the Netherlands; 3 Department of Neuroscience, Erasmus Medical Center, 3015GD Rotterdam, the Netherlands

**Keywords:** Cellular neuroscience, Cell biology, Developmental biology

## Abstract

With the recent findings that mutations in the gene encoding the α-subunit of calcium/calmodulin-dependent protein kinase II (CAMK2A) causes a neurodevelopmental disorder (NDD), it is of great therapeutic relevance to know if there exists a critical developmental time window in which CAMK2A needs to be expressed for normal brain development, or whether expression of the protein at later stages is still beneficial to restore normal functioning. To answer this question, we generated an inducible *Camk2a* mouse model, which allows us to express CAMK2A at any desired time. Here, we show that adult expression of CAMK2A rescues the behavioral and electrophysiological phenotypes seen in the *Camk2a* knock-out mice, including spatial and conditional learning and synaptic plasticity. These results suggest that CAMK2A does not play a critical irreversible role in neurodevelopment, which is of importance for future therapies to treat CAMK2A-dependent disorders.

## Highlights

• Generation of an novel mouse model to induce CAMK2A expression in adult mice
• Adult CAMK2A expression restores all behavior deficits seen in CAMK2A knockout mice
• Adult CAMK2A expression normalizes hippocampal synaptic plasticity
• *Camk2a* is the first NDD-associated gene to show full rescue upon adult reinstatement

## Graphical abstract

**Figure:**
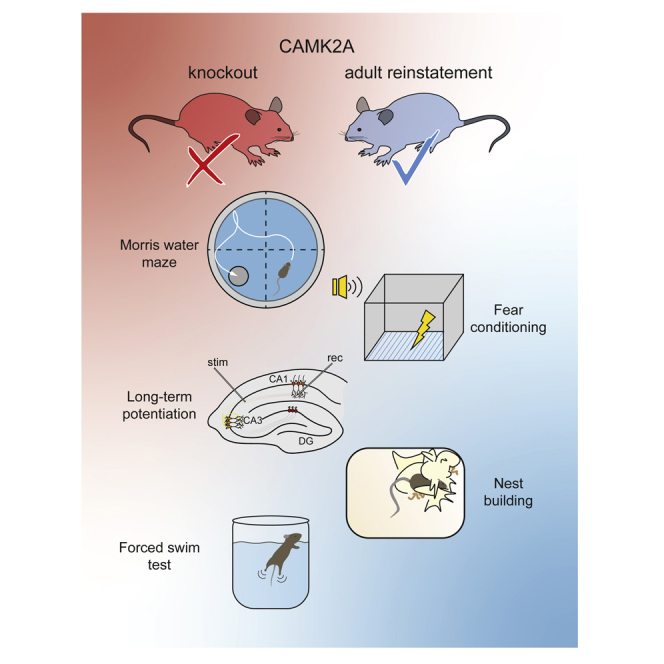

## Introduction

Intellectual disability (ID), a condition defined by an IQ below 70, is a predominant feature of neurodevelopmental disorders (NDDs) affecting approximately 1% of the global world population (Maulik et al., 2011; McKenzie et al., 2016). Because of next-generation sequencing, many genes have now been implicated in this clinical condition (Gilissen et al., 2014; Vissers et al., 2016). One of the genes recently implicated in ID is *CAMK2A*, which encodes the alpha-subunit of calcium/calmodulin-dependent protein kinase II (CAMK2A), a highly abundant kinase in the brain that has been shown to play a critical role in hippocampus-dependent synaptic plasticity, learning, and memory in mice (Silva et al., 1992a, 1992b). In humans, a similar role for CAMK2A has been suggested, as all individuals carrying mutations in *CAMK2A* suffer from a NDD with mild to severe ID, and some from autism spectrum disorder (Akita et al., 2018; Chia et al., 2018; Küry et al., 2017).

With the diagnosis for the *CAMK2A*-dependent NDD established, doors open toward identifying a potential therapy. However, the important question that emerges is whether the genetic mutation causes alterations during early neurodevelopment that might be irrevocable, and thus dictate a critical window for therapeutic intervention (Krol and Feng, 2018; Meredith et al., 2012). The current literature shows that the answer to this question depends strongly on the gene involved in the NDD and the behavior tested (Aceti et al., 2015; Clement et al., 2012; Guy et al., 2007; Lang et al., 2014; Mei et al., 2016; Mielnik et al., 2020; Silva-Santos et al., 2015). For example, in a mouse model for Rett syndrome, reinstatement of the gene *Mecp2* in adulthood improves all phenotypic behavior, but seldom back to wild type performance (Guy et al., 2007; Lang et al., 2014). However, in other models for NDDs, the ability to rescue phenotypes was also shown to depend on the behavior tested, as exemplified by *Grin1*, *Shank3*, *Ube3a*, and *Syngap1* (Aceti et al., 2015; Clement et al., 2012; Mei et al., 2016; Mielnik et al., 2020; Silva-Santos et al., 2015). Social and cognitive deficits are largely, but not completely rescued upon adult reinstatement of *Grin1* (Mielnik et al., 2020) and fully rescued upon *Shank3* reinstatement (Mei et al., 2016), but motor and anxiety deficits are only partially rescued upon *Grin1* reinstatement (Mielnik et al., 2020) and not rescued upon *Shank3* reinstatement (Mei et al., 2016). In a mouse model for Angelman Syndrome, adult reinstatement of *Ube3a* does not rescue behavioral phenotypes found in the *Ube3a* mouse (Silva-Santos et al., 2015). However, reinstatement of *Ube3a* at postnatal day 21 (P21) rescues the motor skill deficits on the rotarod test, but not deficits in anxiety-related or intrinsic behavior, suggesting an early critical window for these forms of behavior (Silva-Santos et al., 2015). In a *Syngap1* haploinsufficiency model, cognitive behavior cannot be rescued upon adult reinstatement, whereas motor and anxiety-related behavior can only be rescued upon very young reinstatement, at P1 but not P21 (Aceti et al., 2015; Clement et al., 2012). Interestingly, pharmacological treatment with a GSK3-β inhibitor of the *Syngap1* mouse model during the critical period of P10-16 does rescue social, cognitive, and anxiety-related behavior, and partially motor behavior (Verma et al., 2021). Finally, the only phenotype that could be rescued upon adult reinstatement in all NDD mouse models discussed here is the electrophysiological plasticity (Guy et al., 2007; Mei et al., 2016; Ozkan et al., 2014; Rotaru et al., 2018; Silva-Santos et al., 2015).

CAMK2A starts to be expressed around P1, which means that it is not required for prenatal neurodevelopment (Bayer et al., 1999). Whereas in the adult brain CAMK2A has been shown to play a critical role in normal brain functioning, as adult deletion of *Camk2a* is equally detrimental for learning and plasticity as germline deletion (Achterberg et al., 2014), not much is known about a possible critical period for CAMK2A during the early postnatal development and whether the phenotypes seen in adult mice could potentially be rescued upon CAMK2A expression.

To assess whether there is a role for CAMK2A in postnatal neurodevelopment and if a critical period for CAMK2A expression exists, we generated an inducible *Camk2a* mouse model, in which we can reinstate *Camk2a* at the time of our choosing. We show that adult reinstatement of *Camk2a* rescues all behavioral and electrophysiological phenotypes seen in the *Camk2a* knockout mice. These results indicate that absence of CAMK2A during development does not lead to irreversible alterations in brain development, and that potential therapies for CAMK2A-related disorders do not require specific early time windows.

## Results

### Generation of an inducible Camk2a mouse

**Figure 1:**
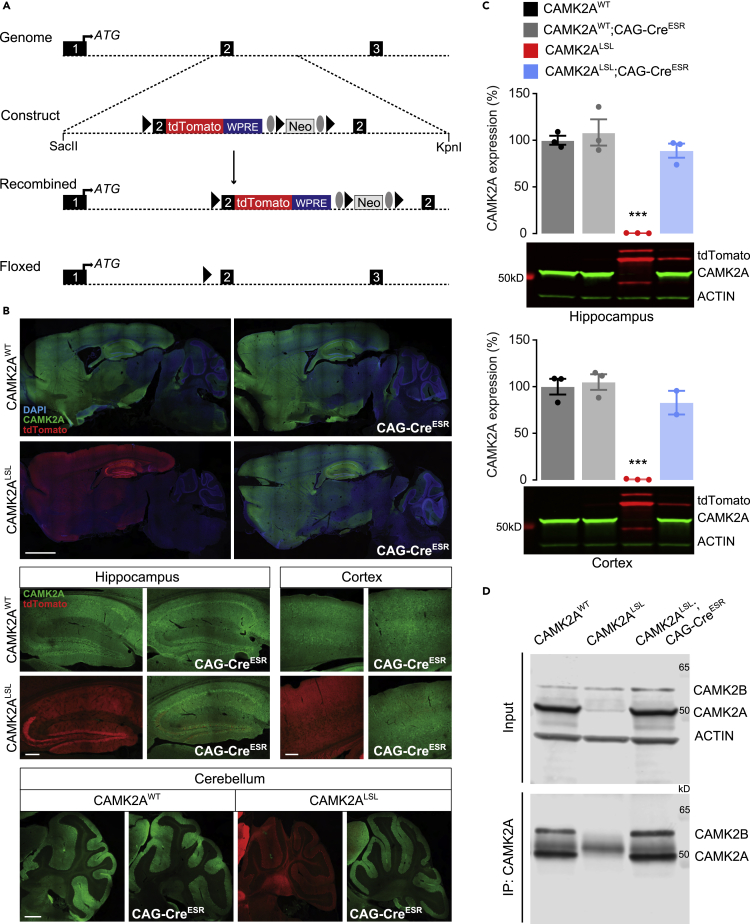
Generation and molecular analysis of the inducible CAMK2A^LSL^;CAG-Cre^ESR^ knock-in mouse (A) To generate CAMK2A^LSL^;CAG-Cre^ESR^ mice, a transgenic cassette was generated in which the *tdTomato* open reading frame was fused to exon 2 of the *Camk2a* gene followed by the woodchuck hepatitis virus post-transcriptional regulatory element (WPRE) flanked by *LoxP* sites and a neomycin cassette, flanked by Frt sites, for positive selection. This cassette was inserted in the *Camk2a* gene in the genome through homologous recombination, as indicated in the schematic overview, which results in *tdTomato* expression under the control of the *Camk2a* promotor. Breeding with CAG-Cre^ESR^ line resulted in floxed *LoxP* sites and transcription of the *Camk2a* gene. Triangles indicate *LoxP* sites and ovals indicate Frt sites. Restriction sites used for linearization of the plasmid are indicated. (B) Representative images of immunofluorescent stainings for CAMK2A and tdTomato on sagittal slices of whole brain (top; scale bar represents 2 mm), hippocampus, and cortex (middle; scale bar represents 200 μm), and cerebellum (bottom; scale bar represents 500 μm). (C) Immunoblot analysis of hippocampal and cortical tissue probed with CAMK2A, tdTomato, and ACTIN antibodies revealed significant CAMK2A reinstatement in the CAMK2A^LSL^;CAG-Cre^ESR^ mice (one-way ANOVA with Bonferroni’s post-hoc analysis. Hippocampus: *F*_(3,8)_ = 36.09, *p* < 0.0001, CAMK2A^WT^ vs CAMK2A^WT^;CAG-Cre^ESR^*p* > 0.999, CAMK2A^WT^ vs CAMK2A^LSL^*p* = 0.0002, CAMK2A^WT^ vs CAMK2A^LSL^;CAG-Cre^ESR^*p* > 0.999, CAMK2A^LSL^ vs CAMK2A^LSL^;CAG-Cre^ESR^*p* = 0.0004. Cortex: *F*_(3,7)_ = 41.85, *p* < 0.0001, CAMK2A^WT^ vs CAMK2A^WT^;CAG-Cre^ESR^*p* > 0.999, CAMK2A^WT^ vs CAMK2A^LSL^*p* = 0.0002, CAMK2A^WT^ vs CAMK2A^LSL^;CAG-Cre^ESR^*p* > 0.999, CAMK2A^LSL^ vs CAMK2A^LSL^;CAG-Cre^ESR^*p* = 0.0013). (D) Immunoprecipitation of CAMK2A on cortical tissue of CAMK2A^WT^, CAMK2A^LSL^, and CAMK2A^LSL^;CAG-Cre^ESR^ mice. Representative blots probed with antibodies against CAMK2A, CAMK2B, and ACTIN are shown. Band of IgG heavy chain is detectable in IP blot between the CAMK2A and CAMK2B bands. See also Figure S1. Data represents mean ± SEM; *n* = 2–3 per group; ∗∗∗*p* < 0.001.

To study the role of CAMK2A during neurodevelopment, we generated a novel mouse model. A transgenic cassette containing *Camk2a* exon 2 fused to the *tdTomato* gene with a transcriptional stop at the end, flanked with *loxP* sites, was inserted between exon 1 and 2 of the endogenous *Camk2a* gene (referred to as CAMK2 Lox – Stop – Lox (CAMK2^LSL^)), allowing for temporally controlled re-expression of *Camk2a* upon Cre-mediated deletion of the transgenic cassette. Heterozygous CAMK2^LSL^ mice were then crossed with the transgenic tamoxifen-inducible CAG-Cre^ESR^ line, to obtain both the CAMK2^LSL^ and the inducible CAMK2A^LSL^;CAG-Cre^ESR^ mouse line (Figure 1A). As control, wild-type littermates negative or positive for Cre-expression were taken along (CAMK2A^WT^ and CAMK2A^WT^;CAG-Cre^ESR^, respectively).

In the absence of Cre-driven recombination, CAMK2A expression in adult CAMK2A^LSL^ mice is abolished and replaced by tdTomato expression driven by the endogenous CAMK2 promotor (Figure 1B). Administration of tamoxifen (0.1 mg/mL, four consecutive days) to adult mice (>8 weeks old), successfully deleted the tdTomato transgene and induced CAMK2A re-expression throughout the brain (Figure 1B). To get an insight into the level of CAMK2A protein expression upon reinstatement, a western blot was performed on hippocampal and cortical tissue of the mice 3–4 weeks after gene reinstatement. Western blot analysis revealed 89% and 83% of CAMK2A expression upon reinstatement in the hippocampus and cortex, respectively, of CAMK2A^LSL^;CAG-Cre^ESR^ mice, with low levels of tdTomato still present (Figure 1C; all statistics are given in the figure legends). Full blots are displayed in Figure S1.

CAMK2A is known to form heteromeric holoenzyme complexes with CAMK2B consisting of 12–14 subunits (Bhattacharyya et al., 2016; Shen et al., 1998). As we started to express CAMK2A here in the adult brain, having the mice to develop with only CAMK2B present, we tested whether adult expressed CAMK2A forms heteromeric holoenzymes again with CAMK2B. Four weeks after tamoxifen injections, we immunoprecipitated CAMK2A from cortex tissue and immunoblotted for both isoforms. CAMK2B was successfully pulled down in CAMK2A^WT^ and CAMK2A^LSL^;CAG-Cre^ESR^ mice, but not in CAMK2A^LSL^, indicating correct formation of heteromeric holoenzymes consisting of CAMK2A and CAMK2B subunits, upon *Camk2a* reinstatement (Figures 1D and S1). Note that the input showed similar CAMK2B expression in all groups, confirming previous findings (Elgersma et al., 2002) that loss of CAMK2A does not result in upregulation of CAMK2B.

### Adult Camk2a reinstatement rescues learning and plasticity phenotypes

**Figure 2:**
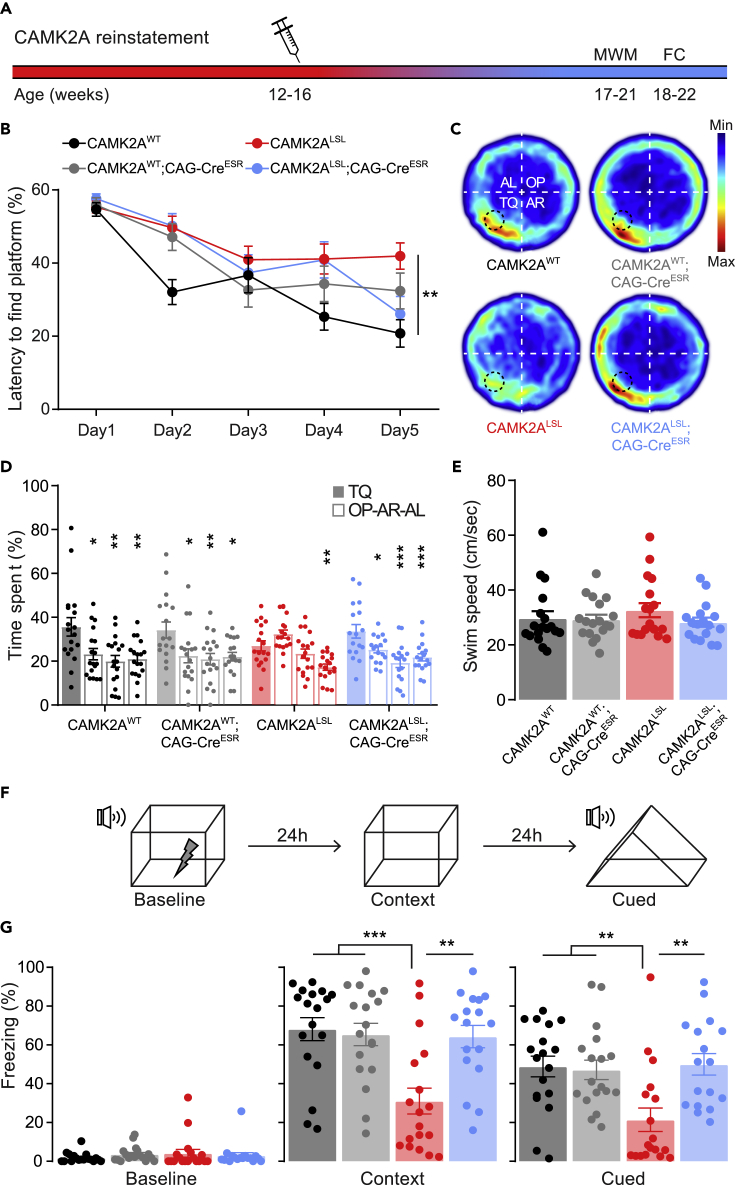
Adult CAMK2A expression rescues cognitive spatial and associative learning (A) Timeline representing reinstatement of CAMK2A in adulthood and behavioral testing, MWM = Morris water maze, FC = fear conditioning. Injection needle indicates tamoxifen injections on four consecutive days. (B) Latency to find the platform over five consecutive days, with two sessions per day (two-way repeated measures ANOVA with Bonferroni’s post-hoc analysis on genotype. Interaction time x genotype: *F*_(12,268)_ = 2.44, *p* = 0.005; genotype: *F*_(3,67)_ = 3.919, *p* = 0.0122, CAMK2A^WT^ vs CAMK2A^WT^;CAG-Cre^ESR^*p* = 0.4251, CAMK2A^WT^ vs CAMK2A^LSL^*p* = 0.0085, CAMK2A^WT^ vs CAMK2A^LSL^;CAG-Cre^ESR^*p* = 0.1296, CAMK2A^WT^;CAG-Cre^ESR^ vs CAMK2A^LSL^*p* = 0.8402, CAMK2A^LSL^ vs CAMK2A^LSL^;CAG-Cre^ESR^*p* > 0.999). (C) Heatmap for probe trial on day 5 indicates where the mice spent most time in the Morris water maze for each genotype. For analysis, the arena was divided into four quarters with the platform in the target quadrant (TQ). OP = opposite, AR = adjacent right, and AL = adjacent left quadrant. (D) Time spent in each quadrant (TQ, OP, AR and AL, respectively) during probe trial on day 5, asterisks indicate differences compared with time spent in TQ within genotype (one-way ANOVA with Bonferroni’s post-hoc analysis. CAMK2A^WT^: *F*_(3,68)_ = 5.94, *p* = 0.0012, TQ vs OP *p* = 0.0128, TQ vs AR *p* = 0.0012, TQ vs AL *p* = 0.0027. CAMK2A^WT^;CAG-Cre^ESR^: *F*_(3,68)_ = 4.62, *p* = 0.0053, TQ vs OP *p* = 0.0166, TQ vs AR *p* = 0.0055, TQ vs AL *p* = 0.0127. CAMK2A^LSL^: *F*_(3,68)_ = 10.46, *p* < 0.0001, TQ vs OP *p* = 0.171, TQ vs AR *p* = 0.600, TQ vs AL *p* = 0.0024. CAMK2A^LSL^;CAG-Cre^ESR^: *F*_(3,64)_ = 8.20, *p* = 0.0001, TQ vs OP *p* = 0.0262, TQ vs AR *p* < 0.0001, TQ vs AL *p* = 0.0009). (E) Swimming speed during probe trial (one-way ANOVA, *F*_(3,67)_ = 0.74, *p* = 0.530). (F) Schematic overview of the fear conditioning procedure. (G) Amount of time the mice spent freezing in the baseline, context, and cued conditions (one-way ANOVA with Bonferroni’s post-hoc analysis. Baseline: *F*_(3,67)_ = 0.52, *p* = 0.671. Context: *F*_(3,67)_ = 8.47, *p* < 0.0001, CAMK2A^WT^ vs CAMK2A^WT^;CAG-Cre^ESR^*p* > 0.999, CAMK2A^WT^ vs CAMK2A^LSL^*p* = 0.0003, CAMK2A^WT^ vs CAMK2A^LSL^;CAG-Cre^ESR^*p* > 0.999, CAMK2A^LSL^ vs CAMK2A^LSL^;CAG-Cre^ESR^*p* = 0.0016. Cued; F_(3,67)_ = 6.24, *p* = 0.0008, CAMK2A^WT^ vs CAMK2A^WT^;CAG-Cre^ESR^*p* > 0.999, CAMK2A^WT^ vs CAMK2A^LSL^*p* = 0.0041, CAMK2A^WT^ vs CAMK2A^LSL^;CAG-Cre^ESR^*p* > 0.999, CAMK2A^LSL^ vs CAMK2A^LSL^;CAG-Cre^ESR^*p* = 0.0031). Data represents mean ± SEM, *n* = 17–18 mice per group. ∗*p* < 0.05, ∗∗*p* < 0.01, ∗∗∗*p* < 0.001.

*Camk2a* knockout mice are known to have severe impairments in spatial as well as associative learning (Chen et al., 1994; Elgersma et al., 2002; Silva et al., 1992a). To confirm that our CAMK2A^LSL^ mice behave as a true knockout, and whether adult gene reinstatement can rescue these phenotypes, 12–16-week-old mice were injected with tamoxifen and, after 5 weeks, spatial and associative learning was assessed using the Morris water maze and contextual and cued fear conditioning (Figure 2A).

Mice were trained to find the hidden platform of the Morris water maze for 5 days. Although latencies to find the platform decreased (with some fluctuations) over time for all genotypes, the overall reduction in latency to find the platform was less in CAMK2A^LSL^ mice compared with CAMK2A^WT^, suggesting potentially impaired procedural learning (Figure 2B). Latencies to find the platform are not very reliable to assess spatial learning, as just missing the platform could immediately lead to longer latencies. To better test whether the mice had used a spatial learning strategy to learn the platform position, we performed a probe trial, removing the platform from the water bath. CAMK2A^WT^ and CAMK2A^WT^;CAG-Cre^ESR^ mice showed clear preferences to spend time in the target quadrant, proving they successfully learned the location of the platform (Figures 2C and 2D). In contrast, CAMK2A^LSL^ mice did not show any preference for the target quadrant confirming impaired spatial learning. Mice in which the *Camk2a* gene was reinstated during adulthood (CAMK2A^LSL^;CAG-Cre^ESR^) showed clear preference for the target quadrant, equivalent to CAMK2A^WT^ mice, showing that spatial learning was normal upon adult *Camk2a* gene reinstatement (Figure 2D). Importantly, the swim speed was similar between genotypes (Figure 2E).

For the fear conditioning paradigm, on day 1, mice were allowed to explore the fear conditioning box for 150 s after which they were exposed for 20 s to a tone, which ended simultaneously with a 2 s mild foot shock. After 24 h, on day 2, mice were placed back in the same box and contextual memory was assessed by measuring the amount of time the mice showed freezing behavior (Figure 2F). Confirming the previously published impaired associative learning (Achterberg et al., 2014; Chen et al., 1994), CAMK2A^LSL^ mice spent significantly less time freezing compared with the other genotypes (Figure 2G). On day 3, mice were placed in a different context and were presented with the tone to test for amygdala-dependent cued learning. Again, CAMK2A^LSL^ mice showed impaired associative learning, spending significantly less time freezing compared with the other genotypes. Similarly as in the Morris water maze experiment, adult expression of CAMK2A induced a full rescue of the associative learning phenotype, as CAMK2A^LSL^;CAG-Cre^ESR^ mice showed equal freezing time compared with CAMK2A^WT^ mice both in contextual and cued fear conditioning (Figure 2G).

**Figure 3:**
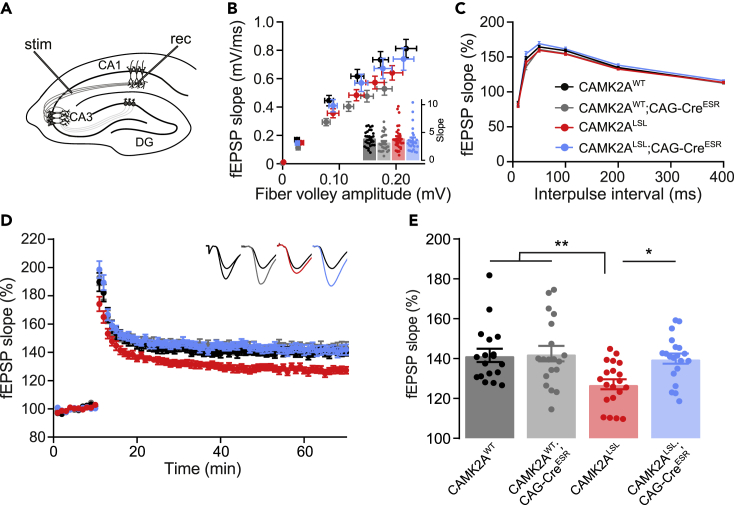
Adult CAMK2A expression rescues synaptic long-term plasticity (A) Schematic overview of the placement of the electrodes for the extracellular field recordings done in Schaffer collateral-CA1 synapses of hippocampal slices. (B) Basal synaptic transmission analysis recorded after increasing stimulation strength (10–100 mA; two-way repeated measures ANOVA with Bonferroni’s post-hoc analysis on genotype. Fiber volley: interaction time x genotype: *F*_(15,540)_ = 1.40, *p* = 0.1401; genotype: *F*_(3,108)_ = 0.55, *p* = 0.6481. fEPSP: interaction time x genotype: *F*_(15,540)_ = 4.18, *p* < 0.0001; genotype: *F*_(3,108)_ = 4.01, *p* = 0.0095, CAMK2A^WT^ vs CAMK2A^WT^;CAG-Cre^ESR^*p* = 0.0001, CAMK2A^WT^ vs CAMK2A^LSL^*p* = 0.0544, CAMK2A^WT^ vs CAMK2A^LSL^;CAG-Cre^ESR^*p* > 0.999, CAMK2A^LSL^ vs CAMK2A^LSL^;CAG-Cre^ESR^*p* = 0.867). Inset shows the slope of the input/output graph (one-way ANOVA, F_(3,108)_ = 1.00, *p* = 0.3976). (C) Paired-pulse facilitation, a form of presynaptic-dependent short-term plasticity (one-way ANOVA with Bonferroni’s post-hoc analysis calculated per interval. 10 ms: *F*_(3,107)_ = 0.16, *p* = 0.922; 25 ms: *F*_(3,107)_ = 5.34, *p* = 0.0018, CAMK2A^WT^ vs CAMK2A^WT^;CAG-Cre^ESR^*p* > 0.999, CAMK2A^WT^ vs CAMK2A^LSL^*p* > 0.999, CAMK2A^WT^ vs CAMK2A^LSL^;CAG-Cre^ESR^*p* > 0.0686, CAMK2A^LSL^ vs CAMK2A^LSL^;CAG-Cre^ESR^*p* = 0.0012; 50 ms: *F*_(3,107)_ = 2.88, *p* = 0.0393, CAMK2A^WT^ vs CAMK2A^WT^;CAG-Cre^ESR^*p* > 0.999, CAMK2A^WT^ vs CAMK2A^LSL^*p* > 0.999, CAMK2A^WT^ vs CAMK2A^LSL^;CAG-Cre^ESR^*p* > 0.0572, CAMK2A^LSL^ vs CAMK2A^LSL^;CAG-Cre^ESR^*p* = 0.1103; 100 ms: *F*_(3,107)_ = 2.35, *p* = 0.0763; 200 ms: *F*_(3,107)_ = 2.09, *p* = 0.1058; 400 ms: *F*_(3,107)_ = 1.18, *p* = 0.3210). (D) Normalized fEPSP slope after induction of LTP with 100-Hz stimulation over time, insets show representative responses before (black) and after (colored) LTP induction. (E) Mean normalized fEPSP slope in the final 10 min of recording (one-way ANOVA with Bonferroni’s post-hoc analysis, F_(3,73)_ = 5.55, *p* = 0.0017, CAMK2A^WT^ vs CAMK2A^WT^;CAG-Cre^ESR^*p* > 0.999, CAMK2A^WT^ vs CAMK2A^LSL^ p = 0.0090, CAMK2A^WT^ vs CAMK2A^LSL^;CAG-Cre^ESR^*p* > 0.999, CAMK2A^LSL^ vs CAMK2A^LSL^;CAG-Cre^ESR^*p* = 0.0221). Data represents mean ± SEM, *n* = 23–30 slices of five to six mice for each group. ∗*p* < 0.05, ∗∗*p* < 0.01.

It is generally accepted that synaptic plasticity, i.e. the strengthening of synapses, underlies learning and memory (Shaw, 1986). Indeed, long-term potentiation (LTP) at the Schaffer collateral-CA1 synapse in the hippocampus is known to be impaired in *Camk2a* knockout mice (Elgersma et al., 2002; Hinds et al., 1998; Silva et al., 1992b). With all the cognitive phenotypes being rescued upon adult *Camk2a* gene reinstatement, we hypothesized that also synaptic plasticity would be rescued upon adult reinstatement. We first measured basal synaptic transmission at the Schaffer collateral-CA1 synapse, where the CAMK2A^LSL^ showed similar strength compared with CAMK2A^WT^ (Figures 3A and 3B). As we found a small Cre effect in the fEPSP in CAMK2A^WT^ mice (Figure 3B), we analyzed the slopes of the input/output curve, to assess the strength of basal transmission, which was similar between all groups (Figure 3B inset). Also paired pulse facilitation (PPF) was found to be similar between all groups (Figure 3C). Upon the induction of LTP, we found, consistent with previous findings (Elgersma et al., 2002; Silva et al., 1992b), a significant impairment in hippocampal LTP in the CAMK2A^LSL^ mice compared with the other genotypes. Moreover, confirming our hypothesis, LTP was completely normalized in the CAMK2A^LSL^;CAG-Cre^ESR^ mice, proving a rescue also of synaptic plasticity upon adult *Camk2a* gene reinstatement (Figures 3D and 3E).

### Adult Camk2a reinstatement rescues intrinsic behavioral phenotypes

**Figure 4:**
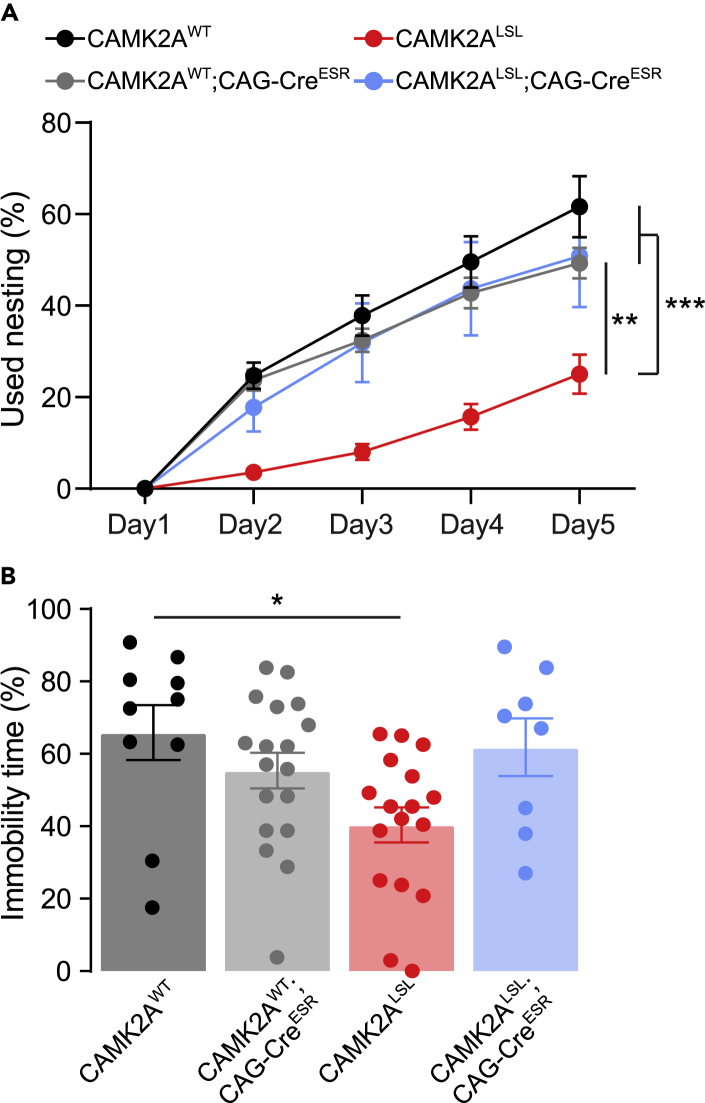
Adult CAMK2A expression rescues intrinsic behavior in nest building and forced swim test (A) Amount of nest material used each day during nest building test over 5 days (two-way repeated measures ANOVA with Bonferroni’s post-hoc analysis on genotype. Interaction time x genotype: *F*_(12,196)_ = 8.79, *p* < 0.0001; genotype: *F*_(3,49)_ = 12.58, *p* < 0.0001, CAMK2A^WT^ vs CAMK2A^WT^;CAG-Cre^ESR^*p* > 0.999, CAMK2A^WT^ vs CAMK2A^LSL^*p* < 0.0001, CAMK2A^WT^ vs CAMK2A^LSL^;CAG-Cre^ESR^*p* > 0.999, CAMK2A^LSL^ vs CAMK2A^LSL^;CAG-Cre^ESR^*p* = 0.0020). (B) Amount of time mice spent immobile in forced swim test (one-way ANOVA with Bonferroni’s post-hoc analysis, *F*_(3,49)_ = 3.68, *p* = 0.0182, CAMK2A^WT^ vs CAMK2A^WT^;CAG-Cre^ESR^*p* > 0.999, CAMK2A^WT^ vs CAMK2A^LSL^*p* = 0.0267, CAMK2A^WT^ vs CAMK2A^LSL^;CAG-Cre^ESR^*p* > 0.999, CAMK2A^WT^;CAG-Cre^ESR^ vs CAMK2A^LSL^*p* = 0.2649, CAMK2A^LSL^ vs CAMK2A^LSL^;CAG-Cre^ESR^*p* = 0.1233). Data represents mean ± SEM, *n* = 8–18 mice per group. ∗*p* < 0.05, ∗∗*p* < 0.01, ∗∗∗*p* < 0.001.

Most of the phenotypes tested above depend on hippocampal plasticity, which appears to not have a critical developmental window, but remains important throughout life (Guy et al., 2007; Ozkan et al., 2014; Silva-Santos et al., 2015). Hence, we also wanted to assess the role of CAMK2A in more intrinsic behavior, which has been shown to have an early critical developmental time window during which alterations can cause irreversible damage (Silva-Santos et al., 2015). For this purpose, we performed a behavioral battery of different tests, previously shown to be sensitive to a critical treatment window to obtain full reversal (Silva-Santos et al., 2015; Sonzogni et al., 2018, 2019, 2020). Similar to the previous experiments, gene reinstatement in this cohort was induced in adult 11–19-week-old mice, and after 5 weeks, a behavioral battery was performed. No behavioral phenotype was found in CAMK2A^LSL^ compared with CAMK2A^WT^ mice in the open field, rotarod, or marble burying test (Figure S2). However, in both the nest building and forced swim test, we found CAMK2A^LSL^ mice to show significantly altered behavior compared with CAMK2A^WT^ mice. In the nest building paradigm, CAMK2A^LSL^ mice used less material to build their nest compared with the other genotypes and in the forced swim test, CAMK2A^LSL^ mice showed decreased immobility compared with CAMK2A^WT^ mice (Figures 4A and 4B). Surprisingly, both phenotypes were rescued in the CAMK2A^LSL^;CAG-Cre^ESR^ mice as they showed similar behavior compared with CAMK2A^WT^ mice, suggesting that although the absence of CAMK2A during early developmental periods affects these intrinsic behaviors, adult CAMK2A expression is beneficial to rescue these behaviors (Figures 4A and 4B). This could indicate that the impaired circuits that underlie the nest building and forced swim test deficits in *Camk2a* mice are different from the circuits that underlie these deficits in *Ube3a* mice (Silva-Santos et al., 2015).

## Discussion

With the current advance in genetic diagnosis, causing a quickly expanding list of rare NDDs and the upcoming possibility to treat these disorders with different forms of gene therapy, such as antisense oligonucleotides, the need to understand the optimal timing for treatment becomes increasingly important. CAMK2-related disorder is one of these recently discovered disorders, and our aim in this study was to understand whether a developmental critical time window exists for CAMK2A to be expressed for normal brain development and function. For this purpose, we generated an inducible CAMK2A knockout model, where we could restore CAMK2A expression in adulthood and assessed the behavior. Together, our results imply that loss of CAMK2A during development does not cause irretrievable distortion to neural circuits in the brain, as we fully rescue the behavioral and electrophysiological phenotypes assessed here.

To our knowledge, this is the only NDD mouse model described in literature, where all phenotypes can be fully normalized upon adult gene reinstatement. In a subset of the NDD mouse models, adult gene reinstatement rescues some but not all behavioral deficits (e.g. upon reinstatement of *Shank3* (Mei et al., 2016)), rescues observed phenotypes partially (e.g. upon reinstatement of *Mecp2* or *Grin1* (Lang et al., 2014; Mielnik et al., 2020)), or only rescues the behavioral deficits if reinstated at a juvenile age (e.g. upon reinstatement of *Ube3a* or *Syngap1* (Aceti et al., 2015; Silva-Santos et al., 2015)). The reverse is also true, as only adult deletion of *Camk2a* completely phenocopies the deficits observed in germline knockout mice (Achterberg et al., 2014), whereas adult deletion in other NDD models do either not or only partially copy the observed behavioral abnormalities (e.g. upon *Syngap1*, *Mecp2*, or *Ube3a* deletion; Gemelli et al., 2006; Ozkan et al., 2014; Sonzogni et al., 2019), or only show the phenotypes upon juvenile deletion (e.g. upon *Ube3a* deletion; Sonzogni et al., 2019), proving a critical window for protein expression of this gene.

The results presented here suggests that CAMK2A does not play a role during development. However, it was recently shown that deletion of *Camk2a* and *Camk2b* simultaneously from germline, is lethal at P1, the moment that CAMK2A starts to be expressed (Kool et al., 2019). With the *Camk2a* and *Camk2b* single knockouts being completely viable, this finding does suggest an important early postnatal developmental role also for CAMK2A, but one that can be compensated for by CAMK2B. Our results now imply that this compensation is of crucial importance for early postnatal brain development, preventing irreversible alterations in the neuronal circuit and therefore allowing for full phenotypic recovery in the adult mice.

To conclude, we show that CAMK2A expression is required during learning or other types of behavior, independent of its expression throughout development. This could be of crucial importance for patients that carry *CAMK2A* loss-of-function mutations (Chia et al., 2018; Küry et al., 2017) and opens doors toward the potential use of therapies that increase CAMK2A expression.

### Limitations of the study

In this study, we generated a novel mouse model, allowing us to assess the effect of expressing CAMK2A in adult mutant mice that lack CAMK2A throughout their life. We found that adult expression of CAMK2A completely normalizes behavior and plasticity in mice. However, we can only draw conclusions on the assays performed in this study. Previously, several additional phenotypes have been reported, such as the cerebellum-dependent vestibular and ocular reflex (VOR and OKR) learning, shown to be affected in CAMK2A knockout mice (Hansel et al., 2006). Similarly, we have only measured plasticity at the CA3-CA1 synapse in the hippocampus. Whether plasticity and other electrophysiological measures shown to be affected in CAMK2A knockout mice (e.g. Coultrap et al., 2014; Hansel et al., 2006; Hardingham et al., 2008) are also normalized, remains to be determined. Finally, we have to be careful in directly translating these findings to the clinic as in our mouse model, CAMK2A is completely absent during development. Most patients, however, express a mutant form of CAMK2A (Akita et al., 2018; Küry et al., 2017).

## STAR★Methods

### Key resources table

**Table:** 

REAGENT or RESOURCE	SOURCE	IDENTIFIER
**Antibodies**		

AffiniPure Fab Fragment donkey anti mouse IgG (H+L)	Jackson ImmunoResearch	Cat# 715-007-003; RRID:AB_2307338
Mouse monoclonal anti-ACTIN	Millipore	Cat# MAB1501R; RRID:AB_2223041
Mouse monoclonal anti-CAMK2A (6G9 clone)	Novus Biologics	Cat# NB100-1983; RRID:AB_10001339
Rabbit polyclonal anti-RFP	Rockland	Cat# 600-401-379; RRID:AB_2209751
Mouse monoclonal anti-CAMK2B (CB-beta-1)	Invitrogen	Cat# 13-9800; RRID:AB_2533045
IRDye 800CW Goat anti-Mouse IgG	LI-COR Biosciences	Cat# 926-32210; RRID:AB_621842
IRDye 680LT Goat anti-Rabbit IgG	LI-COR Biosciences	Cat# 926-68021; RRID:AB_10706309

**Chemicals, peptides, and recombinant proteins**		

Protease inhibitor cocktail		Cat# P8340

**Critical commercial assays**		

Protein A–Agarose Fast Flow beads	Sigma	Cat# P3476
Pierce BCA Protein Assay Kit	Thermo Fisher Scientific	Cat# 23225

**Experimental models: Organisms/strains**		

Mouse: C57Bl6/J	Erasmus MC Rotterdam	
Mouse: 129S2/SvPasCrl	Erasmus MC Rotterdam	
Mouse: 129S2/SvPasCrl Tg(CAG-cre/Esr1∗)5Amc (CAG-Cre^ESR^)		MGI:2182767
Mouse: B6.129 CAMK2^LSL^		MGI:7254975
Mouse: B6.129 CAMK2A^LSL^;CAG-Cre^ESR^	This paper	
Oligonucleotides used for genotyping		
5′ GGATCTGGGCCCCTGTCTGGATC-3′	This paper	
5′ TCTCCCAGGGGTCAAGTTTGGG-3′	This paper	
5′ CTGTCTCCCCGGTCGACATAAC-3′	This paper	

**Recombinant DNA**		

Camk2a-LSL cassette	Erasmus MC (Custom)	

**Software and algorithms**		

EthoVision software	Noldus	https://www.noldus.com/ethovision-xt
VideoFreeze software	Med Associates Inc	https://www.med-associates.com/product/video-fear-conditioning/

### Resource availability

#### Lead contact

Further communication about this manuscript and questions regarding resource availability can be directed to the lead contact Geeske van Woerden (g.vanwoerden@erasmusmc.nl).

#### Materials availability

Mouse lines created in this study have been deposited to Mouse Genome Informatics (MGI: 7254975).

### Experimental model and subject details

#### Generation of mouse line

To generate the CAMK2A^LSL^ mouse model, the construct used to generate the *Camk2a* floxed mouse line was used as a starting point (Achterberg et al., 2014). From this construct the 5′ flanking arm of exon 2 and the 3′ flanking arm containing exon 2 of *Camk2a* were obtained and cloned in a vector containing the cassette of *Camk2a*-exon2 fused to tdTomato, followed by the woodchuck hepatitis virus post-transcriptional regulatory element (WPRE), flanked by *LoxP* sites, and a neomycin cassette for positive selection, flanked by Frt sites. All exonic sequences were sequenced to verify that no mutations were introduced accidentally. The targeting construct was linearized and electroporated into E14 ES cells (derived from 129P2 mice). Cells were cultured in BRL cell-conditioned medium in the presence of leukemia inhibitory factor. After selection with G418 (200 μg/mL), targeted clones were identified by PCR (long-range PCR from neomycin resistance gene to the region flanking the targeted sequence). A clone with normal karyotype was injected into blastocysts of C57BL/6J mice. Male chimeras were crossed with female C57BL/6J mice and resulting offspring was further backcrossed into the C57BL/6J background. To obtain the mice used for experiments, heterozygous CAMK2^LSL^ females (15 times backcrossed in C57BL/6J) were crossed with Tg(CAG-cre/Esr1∗)5Amc (CAG-Cre^ESR^) male mice (MGI:2182767) (17 times backcrossed in 129S2/SvPasCrl). Of the F1 offspring, the heterozygous CAMK2A^LSL^;CAG-cre^ESR^ were crossed with heterozygous CAMK2A^LSL^ mice, obtaining the resulting experimental group: CAMK2A^LSL^, CAMK2A^LSL^;CAG-Cre^ESR^, CAMK2A^WT^ and CAMK2A^WT^;CAG-Cre^ESR^. Adult (>8 weeks) mice of both sexes were used for the experiments. During the experiments, the experimenter was blind for the genotypes. All mice were group housed at 22 ± 2°C, except for the nest building test when they were single caged. They were on a 12/12h light/dark cycle with food and water available *ad libitum*. All experiments were performed during the light phase. All animal experiments were conducted in accordance with the European Commission Council Directive 2010/63/EU (CCD project license AVD101002017893), and all described experiments and protocols were subjected to ethical review (and approved) by an independent review board (IRB) of the Erasmus MC.

### Method details

#### Tamoxifen treatment

To induce Cre-mediated deletion, mice received intraperitoneal injections of 0.1 mg Tamoxifen per gram body weight for four consecutive days at 11–19 weeks old. Tamoxifen was freshly dissolved at a concentration of 20 mg/mL in sunflower oil beforehand.

#### Immunohistochemistry

Adult mice were transcardially perfused with paraformaldehyde (4%) to fixate tissue and brains were extracted. Gelatin-embedded slices of 40 μm thick were blocked in 3% H_2_O_2_ in PBS, rinsed, and for antigen retrieval incubated in sodium citrate (10 mM) at 80°C for 20 min. The slices were again rinsed and placed in blocking solution (10% normal horse serum and 0.5% Triton X-100 in PBS) for 1h at room temperature and incubated overnight at 4°C in blocking solution with AffiniPure Fab Fragment (donkey anti mouse IgG (H+L), 1:200, Cat# 715-007-003, Jackson ImmunoResearch). The next day, slices were incubated for 48–72h with primary antibodies (anti-CAMKII alpha (6G9 clone), 1:100, NB100-1983, Novus Biologics; anti-RFP, 1:5000, 600-401-379, Rockland) shaking delicately at 4°C. Secondary antibodies (Alexa, 1:200, Jackson ImmunoResearch) were incubated for 2h at room temperature. Finally, slices were incubated with 40,6-diamidino-2-phenylindole solution (DAPI, 1:10,000, Invitrogen) for 10 min and mounted in Mowiol medium. Images were acquired with a confocal microscope (LSM700, Zeiss).

#### Western blot

Mice were anesthetized with isoflurane. Cortical and hippocampal tissue was isolated and snap frozen in liquid nitrogen. Samples were sonicated in lysis buffer (0.1 M Tris-HCl [pH 6.8], 4% SDS), containing protease inhibitor cocktail (P8340, Sigma). Protein concentration of the supernatant was determined using the BCA protein assay (Pierce™) and 15 μg was loaded on the gel (3450124, Bio-rad), which was next turbo transferred on nitrocellulose membrane (1704159, Bio-rad). Blots were probed with primary antibodies (anti-CAMKII alpha (6G9 clone), 1:20,000, NB100-1983, Novus Biologics; anti-RFP, 1:2000, 600-401-379, Rockland; anti-ACTIN, 1:10.000, MAB1501R, Millipore) overnight at 4°C. Next day, blots were incubated with IRDye® 800CW Goat anti-Mouse and 680LT Goat anti-Rabbit secondary antibodies (Licor, 1:15,000) for 1 h at room temperature. Blot were imaged using an Odyssey® Imager (Licor) and analyzed using Image Studio™ Lite (Licor).

#### Immunoprecipitation

Cortical tissue was homogenised in lysis buffer (1mM NaHCO_3_, 1 mM MgCl_2_, 0.32 M sucrose, 10 mM HEPES [pH 7.4]) containing protease inhibitor cocktail (P8340, Sigma). Supernatant, containing soluble proteins, was pre-cleared with Protein A agarose beads (P3476, Sigma). An ‘input’ sample was set aside and 900 μL of supernatant was incubated overnight with beads cross-linked to CAMK2A primary antibody (10 μg, NB100-1983, Novus Biologics) rotating end-over-end at 4°C. After washing (150 mM NaCl, 0.1% Triton X-100, 25 mM HEPES [pH 7.4]), protein was eluted by boiling for 5 min at 95°C. Input, unbound and bound IP samples were handled by western blot, which was probed for CAMK2A (1:20,000, NB100-1983, Novus Biologics), CAMK2B (1:10,000, 13-9800, Invitrogen) and ACTIN (1:20,000, MAB1501R, Millipore).

#### Behavior

All experiments were conducted during the light phase of the light/dark cycle and experimenters were blind to genotype. Mice were handled beforehand. Mice that were tested in the Morris water maze, were subsequently tested in fear conditioning. A separate cohort of mice were tested in this order for rotarod, open field, marble burying, nest building and forced swim test.

##### Morris water maze

Mice were trained to find a submerged platform (1 cm submerged, 11 cm in diameter) in a circular pool (1.2 m in diameter) for 5 days. Trials consisted of placing the mouse 30 s on the platform and releasing it at a pseudorandom location in the water maze. If the mouse did not locate the platform within 60 s, the experimenter gently guided the mouse to it. The same procedure was then repeated a second time. Latency to find the platform was manually scored. A probe trial was carried out on day 5 before training, in which the platform was removed after the mouse was initially placed there for 30 s. During the probe trial the mouse was tracked using EthoVision® software (Noldus®). Throughout the experiment the water was made opaque with white paint and kept at a temperature of 25–26°C.

##### Fear conditioning

Mice were placed in a soundproof box (26 × 22 × 18 cm; San Diego Instruments) with a grid floor, white light turned on and a camera monitoring the behavioral activity. The training session consisted of 200 s, wherein after 150 s a conditioned stimulus of a tone (20 s, 85 dB) was presented followed by the unconditioned stimulus of a foot shock via the grid floor (1.0 mA, 2 s) after which the mice were left in the box for the remaining 28 s. 24h later, contextual memory was monitored for 180 s by placing the mice in the same box. Another 24h later, cued memory was monitored for 220 s by placing the mice in an adapted version of the box and after 120 s presenting them with the conditioned stimulus for 100 s. The box was adapted by removing the grid floor, placing plexiglass walls in a triangular shape, turning the light off, and presence of an acetonic odor. Freezing behavior was analyzed with Video Freeze® software (Med Associates Inc) and started when the mouse showed no activity for 1.00 s.

##### Nest building

The mice were single caged 5–7 days before the start of the experiment. Used nest building material was removed from the cages and replaced by 11 g compressed extra-thick blot filter paper (Bio-rad). Every 24h for 5 days, unused material was weighted.

##### Forced swim test

Mice were placed in a glass cylinder (diameter of 18 cm, height of 27 cm) filled with water up to 15 cm (26 ± 1°C) for 6 min and recorded with a camera. After 2 min of habituation, the mice were scored manually for immobility for 4 min. Immobility was defined as no activity other than that needed for the mouse to remain afloat and keep its balance.

##### Open field

Mice were placed in a circular open arena (diameter 110 cm) that was brightly lit for 10 min. The mouse was tracked and distance traveled was measured using EthoVision® software (Noldus®).

##### Marble burying

We equally distributed 20 blue glass marbles on a layer of 4 cm bedding (Lignocel Hygenic Animal Bedding, JRS) in an open Makrolon cage (50 × 26 × 18 cm). Mice were placed inside the cage for 30 min to freely roam and explore the marbles. After the 30 min, marbles were scored manually to be buried when >50% of the marble was covered by bedding.

##### Rotarod

Mice were placed on an accelerating cylinder (4–40 rpm; Ugo Basile Biological Research Apparatus, model 7650) for a maximum of 5 min for 5 consecutive days with 2 trials per day, 45–60 min interval between trials. Latency to fall off the accelerating rotarod was scored manually. When a mouse clung to the cylinder for 3 consecutive rotations this was also scored as the latency to fall off.

#### Electrophysiology

Mice were decapitated under anesthesia of isoflurane and brains were rapidly removed. Sagittal slices of 400 μm were cut with a vibratome (PELCO easiSlicer™, Ted Pella) in ice-cold oxygenated (95%) and carbonated (5%) artificial cerebral spinal fluid (ACSF; in mM: 120 NaCl, 3.5 KCl, 2.5 CaCl2, 1.3 MgSO4, 1.25 NaH2PO4, 26 NaHCO3, and 10 D-glucose). Hippocampi were isolated and allowed to recover in oxygenated and carbonated ACSF at room temperature for 90 min. Slices were placed in a submerged chamber with an ACSF flow of 2 mL/min at 30°C degrees. Bipolar platinum/iridium electrodes (Frederick Haer) were placed in the Shaffer collateral CA1-CA3 region for stimulation and in the dendrites of CA1 pyramidal cells for recording. Slices were left to habituate to the electrode placements for 30 min, before the onset of the experiment. Stimulations were at 1/3^rd^ max fEPSP strength and lasted 100 μs. Paired-pulse facilitation was measured by three rounds of two sequential stimulations at intervals of 10 – 25 – 50 – 100 – 200 – 400 μs. For the LTP experiment, responses were recorded at 1 Hz. After 10 min baseline, LTP was induced by tetanic stimulation of 100 Hz for 1 s and recorded for 60 min. During analysis, responses were normalised to the baseline. Recordings that showed an unstable baseline were excluded.

### Quantification and statistical analysis

Statistical significance was determined using one-way or two-way ANOVA test with Bonferonni post hoc comparison in PRISM software (Graphpad), as data were assumed to be normally distributed. p-Values <0.05 were considered significant. Exact p-values are indicated in the figure legends, CAMK2A^WT^;CAG-Cre^ESR^ vs experimental groups are only represented if the significance is not equal to CAMK2A^WT^. When CAMK2A^LSL^ showed a phenotype compared to CAMK2A^WT^ mice, significance between all groups is indicated in the figures.

## Data Availability

Any additional information required to reanalyse the data reported in this paper is available from the lead contact upon request. Adult (>8 weeks) CAMK2A^LSL^, CAMK2A^LSL^;CAG-Cre^ESR^, CAMK2A^WT^ and CAMK2A^WT^;CAG-Cre^ESR^ mice of both sexes were used for the experiments. During the experiments, the experimenter was blind for the genotypes. All mice were group housed at 22 ± 2°C, except for the nest building test when they were single caged. They were on a 12/12h light/dark cycle with food and water available *ad libitum*. All experiments were performed during the light phase. All animal experiments were conducted in accordance with the European Commission Council Directive 2010/63/EU (CCD project license AVD101002017893), and all described experiments and protocols were subjected to ethical review (and approved) by an independent review board (IRB) of the Erasmus MC. All data reported in this paper will be shared by the lead contact upon request.
