# Supplementary material for: Adult Camk2a gene reinstatement restores the learning and plasticity deficits of Camk2a knockout mice
Source: iScience. 2022 Oct 8;25(11):105303. doi: 10.1016/j.isci.2022.105303 (PMC9593899; doi:10.1016/j.isci.2022.105303)
Supplement: Document S1. Figures S1 and S2 [file mmc1.pdf]

iScience, Volume *25*

Supplemental information

Adult *Camk2a* gene reinstatement restores the learning and plasticity deficits of *Camk2a* knockout mice

Pomme M.F. Rigter, Ilse Wallaard, Mehrnoush Aghadavoud Jolfaei, Jenina Kingma, Laura Post, Minetta Elgersma, Ype Elgersma, and Geeske M. van Woerden

Figure S1

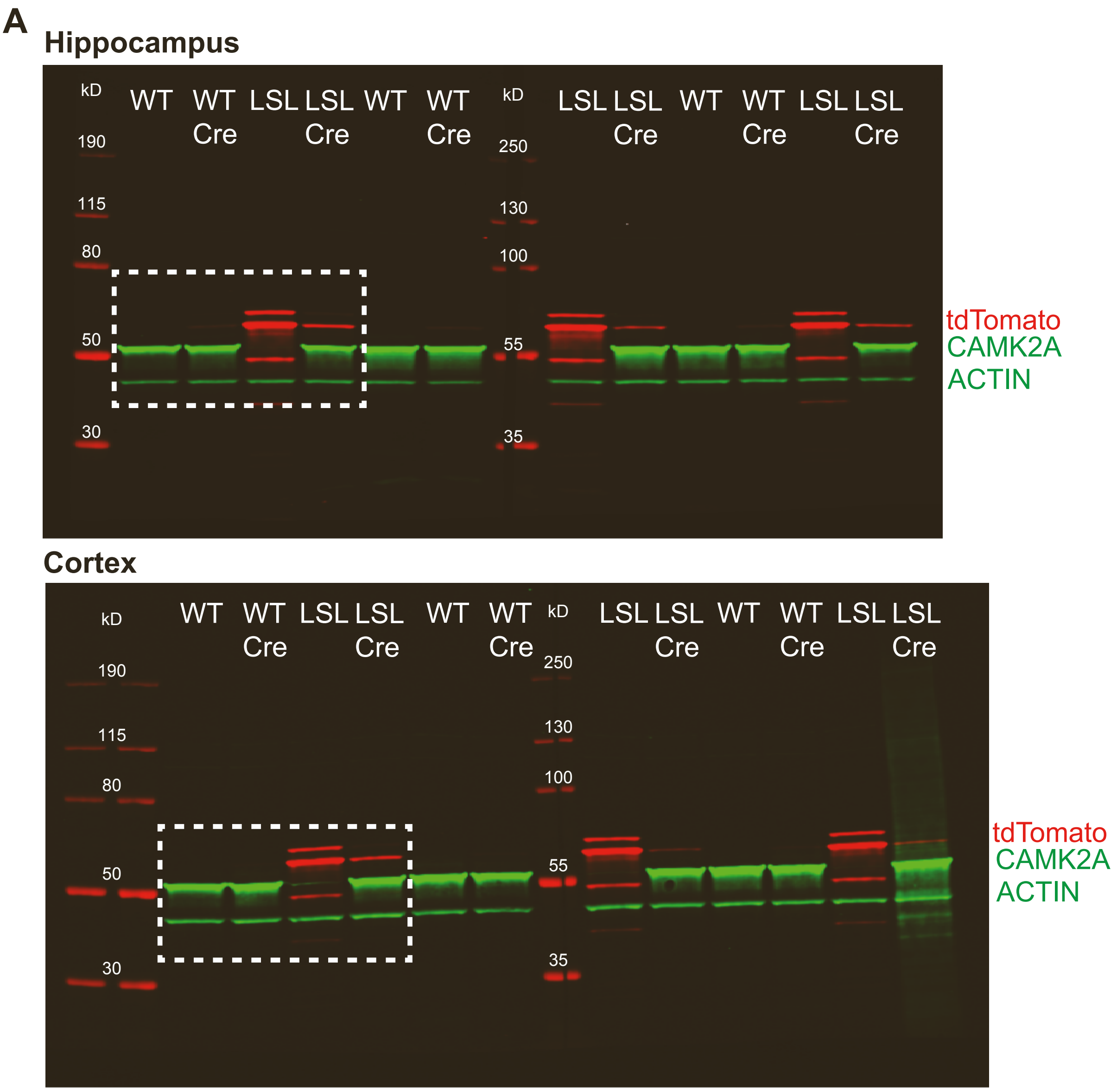

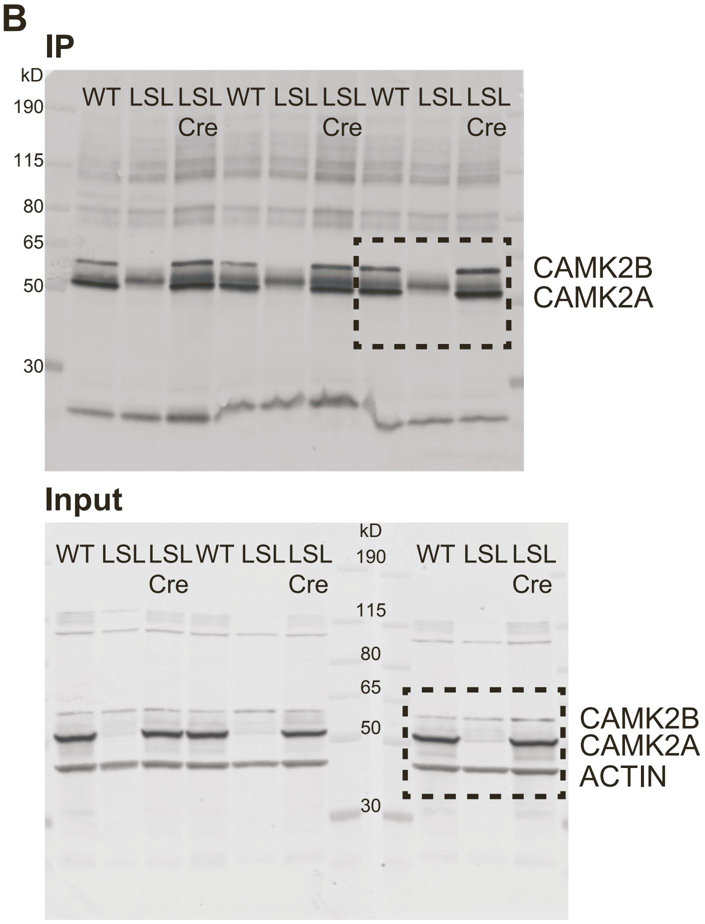

Figure S1: Raw immunoblots, related to Figure 1
Full immunoblots, dashed lines indicate cropped regions that were used for Figure 1. **A)** Blots of hippocampus and cortex probed with CAMK2A, tdTomato and Actin antibodies, related to Figure 1C. **B)** Blots of immunoprecipitation on cortex probed with CAMK2A, CAMK2B and Actin antibodies, related to Figure 1D.

Figure S2

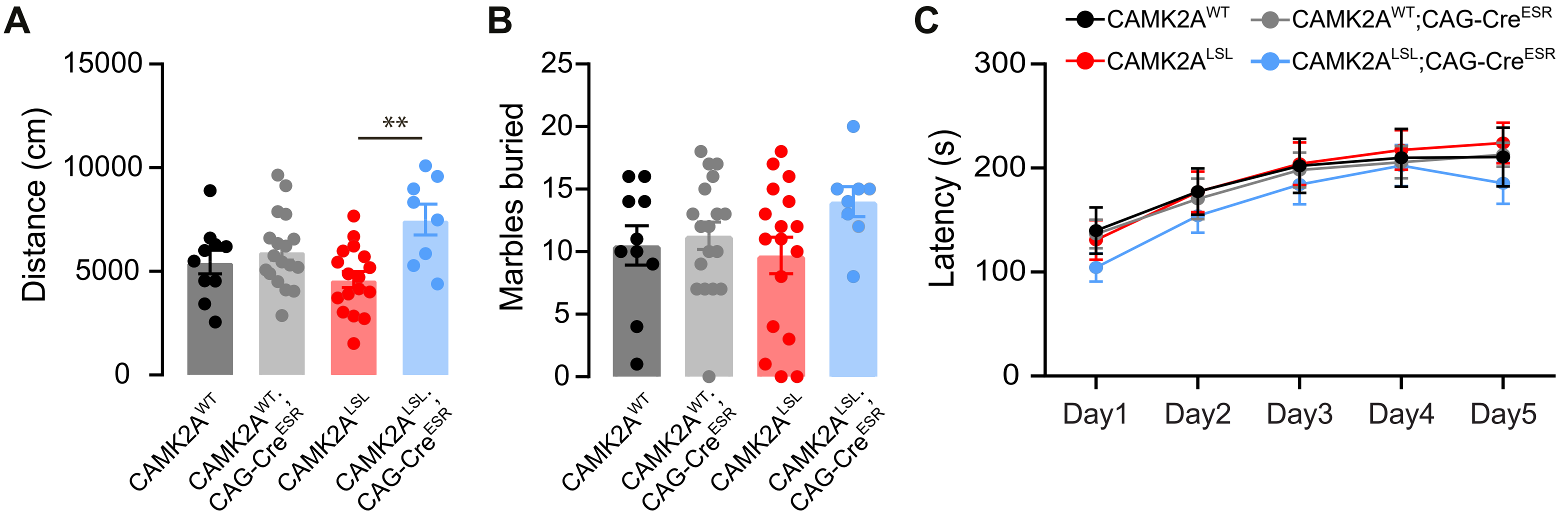

Figure S2: Behavioural tasks in which CAMK2A^{LSL} mice did not show a phenotype, related to Figure 4

A) Distance travelled in the open field task (one-way ANOVA with Bonferroni's post-hoc analysis. Interaction time x genotype: $F_{(3,49)} = 5.06$, $p = 0.0039$; CAMK2A^{WT} vs CAMK2A^{LSL} $p > 0.999$, CAMK2A^{WT} vs CAMK2A^{LSL};CAG-Cre^{ESR} $p = 0.1133$, CAMK2A^{LSL} vs CAMK2A^{LSL};CAG-Cre^{ESR} $p = 0.0025$. **B)** Marbles buried in the marble burying task (one-way ANOVA, $F_{(3,49)} = 1.37$, $p = 0.2631$). **C)** Latency to fall off accelerating rotarod (two-way repeated measures ANOVA. Interaction time x genotype: $F_{(12,196)} = 0.31$, $p = 0.9872$. Genotype: $F_{(3,49)} = 0.28$, $p = 0.8383$). Data represents mean ± SEM, n = 8-18 mice per group, ** $p < 0.01$.
